# Liver toxicity associated with tuberculosis chemotherapy in the REMoxTB study

**DOI:** 10.1186/s12916-018-1033-7

**Published:** 2018-03-28

**Authors:** Conor Duncan Tweed, Genevieve Helen Wills, Angela M. Crook, Rodney Dawson, Andreas H. Diacon, Cheryl E. Louw, Timothy D. McHugh, Carl Mendel, Sarah Meredith, Lerato Mohapi, Michael E. Murphy, Stephen Murray, Sara Murthy, Andrew J. Nunn, Patrick P. J. Phillips, Kasha Singh, M. Spigelman, S. H. Gillespie

**Affiliations:** 1 MRC Clinical Trials Unit at University College London, London, UK; 2 University of Cape Town Lung Institute, Cape Town, South Africa; 3 TASK Applied Science, Cape Town, South Africa; 4 Madibeng Centre for Research, Brits, South Africa; 5 Division of Infection and Immunity, University College London, London, UK; 6 TB Alliance, New York, NY USA; 7 Perinatal HIV Research Unit, Johannesburg, South Africa; 8 Division of Pulmonology, University of San Francisco, San Francisco, USA; 9 The Doherty Institute for Infection and Immunity, University of Melbourne and Royal Melbourne Hospital, Melbourne, Australia; 10 University of St Andrews Medical School, St Andrews, UK

**Keywords:** Tuberculosis, Hepatotoxicity, Drug-induced liver injury, Treatment monitoring

## Abstract

**Background:**

Drug-induced liver injury (DILI) is a common complication of tuberculosis treatment. We utilised data from the REMoxTB clinical trial to describe the incidence of predisposing factors and the natural history in patients with liver enzyme levels elevated in response to tuberculosis treatment.

**Methods:**

Patients received either standard tuberculosis treatment (2EHRZ/4HR), or a 4-month regimen in which moxifloxacin replaced either ethambutol (isoniazid arm, 2MHRZ/2MHR) or isoniazid (ethambutol arm, 2EMRZ/2MR). Hepatic enzymes were measured at 0, 2, 4, 8, 12 and 17 weeks and as clinically indicated during reported adverse events. Patients included were those receiving at least one dose of drug and with two or more hepatic enzyme measurements.

**Results:**

A total of 1928 patients were included (639 2EHRZ/4HR, 654 2MHRZ/2MHR and 635 2EMRZ/2MR). DILI was defined as peak alanine aminotransferase (ALT) ≥ 5 times the upper limit of normal (5 × ULN) or ALT ≥ 3 × ULN with total bilirubin > 2 × ULN. DILI was identified in 58 of the 1928 (3.0%) patients at a median time of 28 days (interquartile range IQR 14–56). Of 639 (6.4%) patients taking standard tuberculosis therapy, 41 experienced clinically significant enzyme elevations (peak ALT ≥ 3 × ULN). On standard therapy, 21.1% of patients aged >55 years developed a peak ALT/aspartate aminotransferase (AST) ≥ 3 × ULN (*p* = 0.01) and 15% of HIV-positive patients experienced a peak ALT/AST ≥ 3 × ULN compared to 9% of HIV-negative patients (*p* = 0.160). The median peak ALT/AST was higher in isoniazid-containing regimens vs no-isoniazid regimens (*p* < 0.05), and lower in moxifloxacin-containing arms vs no-moxifloxacin arms (*p* < 0.05). Patients receiving isoniazid reached a peak ALT ≥ 3 × ULN 9.5 days earlier than those on the ethambutol arm (median time of 28 days vs 18.5 days). Of the 67 Asian patients with a peak ALT/AST ≥ 3 × ULN, 57 (85.1%) were on an isoniazid-containing regimen (*p* = 0.008).

**Conclusions:**

Our results provide evidence of the risk of DILI in tuberculosis patients on standard treatment. Older patients on standard therapy, HIV-positive patients, Asian patients and those receiving isoniazid were at higher risk of elevated enzyme levels. Monitoring hepatic enzymes during the first 2 months of standard therapy detected approximately 75% of patients with a peak enzyme elevation ≥3 × ULN, suggesting this should be a standard of care. These results provide evidence for the potential of moxifloxacin in hepatic sparing.

**Electronic supplementary material:**

The online version of this article (10.1186/s12916-018-1033-7) contains supplementary material, which is available to authorized users.

## Background

The combination of isoniazid, rifampicin, pyrazinamide and ethambutol taken over at least 6 months has been standard therapy for drug-sensitive tuberculosis (TB) for over 30 years [1]. Liver enzyme elevations and drug-induced liver injury (DILI) are recognised complications for this regimen. They cause patient morbidity, treatment interruption and poor treatment outcomes [2–4]. The reported rates of significant liver enzyme elevation on standard therapy vary between 5% and 30% [5–8]. An increased risk is associated with pre-existing liver disease, HIV infection, alcohol use, being female and increasing age [9–12].

Management guidelines for TB DILI are largely based on expert opinion [6, 13, 14]. The British Thoracic Society (BTS) guidelines recommend treatment interruption when alanine aminotransferase (ALT) or aspartate aminotransferase (AST) are elevated to 5 times the upper limit of normal (5 × ULN) or when bilirubin rises above the ULN [13]. The American Thoracic Society (ATS), in contrast, recommends interruption when hepatic enzyme levels are greater than 3 × ULN with symptoms or jaundice, or more than 5 × ULN in asymptomatic patients [6].

The timing and magnitude of elevations in the serum transaminases over the course of treatment in all patients, with and without symptoms, remain unclear, making appropriate management of liver toxicity, including timing of treatment interruption and re-introduction uncertain [6, 15, 16].

To address this evidence gap, we extracted data from a large number of well-characterised patients with the aim of describing the pattern of liver enzyme elevation in standard TB therapy and identifying predisposing factors. We also identified the timing and severity of liver enzyme elevation in two experimental regimens. To our knowledge, this is the largest and most comprehensive available analysis of liver biochemical data for patients taking TB treatment.

## Methods

### REMoxTB database

All data were obtained from the double-blind placebo controlled REMoxTB trial reported previously [17]. Patients were over 18 years old with smear-positive pulmonary TB. The treatment arms were: (i) a regimen of isoniazid (H), rifampicin (R), ethambutol (E) and pyrazinamide (Z) for 8 weeks followed by 18 weeks of isoniazid and rifampicin (2EHRZ/4HR); (ii) a regimen that replaced ethambutol with moxifloxacin for 17 weeks (the isoniazid arm), followed by 9 weeks of placebo (2MHRZ/2MHR) and (iii) a regimen that replaced isoniazid with moxifloxacin for 17 weeks (the ethambutol arm), followed by 9 weeks of placebo (2EMRZ/2MR). ALT or AST > 3 × ULN, total bilirubin > 2.5 × ULN, or Child–Pugh class C liver failure at screening were exclusion criteria. Drug dosing was based on patient weight at screening and can be found in Additional file 1: Table S1.

For this study, baseline characteristics, including the presence or absence of cavities on a chest X-ray, were collected. Liver biochemical tests (LBTs) were collected for safety monitoring at screening and at weeks 2, 4, 8, 12 and 17 with additional tests carried out as required clinically. In our analysis, we included all patients who had received one or more doses of trial medication and had LBTs measured at least twice.

### Laboratory methods and data management

LBTs were performed in local laboratories and the normal ranges for each laboratory were used in this analysis. LBT results from between 14 days before the first dose of medication and 7 days after the last dose of trial medication were included. Results for ALT, AST and total bilirubin were expressed as multiples of the upper limit of the local normal range (ULN).

In this study, DILI was defined as either ALT ≥ 5 × ULN or ALT ≥ 3 × ULN with total bilirubin >2 × ULN, based on the 2011 international DILI Expert Working Group consensus statement [18]. We considered all ALT elevations ≥3 × ULN [with or without bilirubin elevations or increased international normalised ratio (INR)] as clinically significant based on thresholds used by both the ATS and BTS guidance [6, 13]. The trial adverse event reporting system was used to obtain details of symptoms at the time of DILI episodes and clinically significant elevations by collecting all events within a time window of 5 days before through to 5 days after the peak ALT elevation. For patients with a peak ALT ≥ 3 × ULN, serious adverse event narratives for the REMoxTB trial were reviewed for information regarding treatment interruptions, herbal medications, alcohol use and additional tests carried out. Additionally, where sufficient information was available, we applied the Roussel–Uclaf causality assessment method (RUCAM) [19, 20] to identify a causal relationship between the TB medication and peak ALT ≥ 3 × ULN. The criteria for the RUCAM score can be found in Additional file 1: Table S2.

We searched the recorded adverse events for the terms ‘ascites’, ‘cirrhosis’, ‘child’, ‘hepatic’, and ‘liver’ and applied the Child–Pugh scoring criteria (Additional file 1: Table S3) to those patients with evidence of chronic liver disease within 1 month of their first dose of trial medication, to categorise those with stage A or B liver disease.

We identified all patients with ALT > 1 × ULN at baseline and further ALT elevations on treatment. LBTs in the database were labelled as having been taken either at a protocol-scheduled visit or at an unscheduled visit that was clinically indicated. For each treatment arm, we calculated the proportion of LBTs that were taken at scheduled visits compared to unscheduled tests for both DILI cases and clinically significant elevations.

Liver-related withdrawals were defined as patients withdrawn from treatment following a peak ALT or AST ≥ 3 × ULN with only liver-related adverse events (either clinical or relating to biochemical tests) recorded between their peak elevation and the time of withdrawal. Patients who met these criteria were counted for all three treatment arms.

### Statistical methods

Data handling and statistical analysis were carried out using Stata version 13.1 (StataCorp, Texas). A peak value for ALT, AST, bilirubin and INR, along with the time in days from first dose, was identified for each patient. The kappa test statistic was used to assess agreement between ALT and AST.

The Mann–Whitney U test was used to investigate the differences in the distribution of the peak ALT values and the time to reach a peak value. Depending on the number of patients in each group, the chi-squared or Fisher’s exact test (if *n* ≤ 5 in any cell) was used to investigate the differences in peak values when grouped as ≥3 × ULN and <5 × ULN, ≥5 × ULN and <10 × ULN, and ≥10 × ULN. Comparisons made were either pairwise comparing each experimental arm to standard therapy (control), or isoniazid- or moxifloxacin-containing regimens were combined and compared to the remaining treatment regimen to investigate the effect of isoniazid. Kaplan–Meier curves were constructed for the time taken to reach peak ALT and AST for all patients in the trial. We also constructed Kaplan–Meier curves for the time taken to return to within the normal range for patients with a peak ALT or AST of >1 × ULN in all treatment arms. The log-rank test was used to compare the hazard functions between the experimental and standard treatment arms for the events of interest (time to peak and time to normalise) occurring at any time point. Scatter plots were constructed to show the value and timing of elevations in ALT and AST in each treatment arm for patients with a peak value ≥3 × ULN.

## Results

### Liver enzyme results during treatment

Of the 1931 patients randomised in the trial, three patients were excluded from our analysis. A single patient did not receive medication and two patients had only one blood sample taken. Our final analysis population consisted of 639 patients on standard TB therapy, 654 patients allocated to the isoniazid arm and 634 patients on the ethambutol arm. We found 96% agreement (kappa score of 0.58) between the peak ALT and AST results for patients with clinically significant elevations.

The peak value for ALT (0.78 and 0.73 vs 0.83 × ULN) and AST (0.93 and 0.90 vs 1.02 × ULN) was found to be significantly lower in the moxifloxacin-containing arms compared to the standard therapy group (*p* < 0.05). Table 1 shows the peak ALT values and timings in the three treatment arms with details of bilirubin elevations and INR measurements for those patients with peak ALT ≥ 3 × ULN. The range of the median peak value for ALT and AST for all patients varied between 0.73 × ULN to 1.02 × ULN across the treatment arms. The median ALT and AST concentrations for all subjects at scheduled visits over 4 months are illustrated in Additional file 1: Figure S1.

**Table 1:** Summary of the numbers of patients with significant elevations in liver enzyme concentration (≥3 × ULN, ≥5 × ULN and ≥10 × ULN) by treatment arm

	Standard arm (2EHRZ/4HR)	Isoniazid arm (2MHRZ/2MHR)	Ethambutol arm (2EMRZ/2MR)	*p* value
*n* ^1^	634	649	634	
Median peak ALT as × ULN (IQR)	0.83 (0.56–1.35)	0.78 (0.53–1.23)	0.73 (0.51–1.09)	0.046^5^ 0.000^6^
Median time to peak ALT in arm (days)	28 (14–84)	28 (14–84)	55 (14–84)	0.972^5^ 0.017^6^
Median time to peak ALT (days) if ≥3 × ULN	28 (14–56)	18 (14–56)	28 (27–56)	0.755^5^ 0.605^6^
Median time to ALT < 1 × ULN^2^ (days)	26 (15–42)	28 (19–42)	39 (30–61)	0.560^5^ 0.270^6^
No with peak ALT ≥ 3 × ULN and < 5 × ULN (%n^3^)	21 (3.3%)	17 (2.6%)	11 (1.7%)	0.204^7^
Bilirubin > 2 × ULN	2 (0.3%)	4 (0.6%)	0 (0.0%)	0.142^7^
INR^4^ >1.5	0 (0.0%)	3 (0.5%)	1 (0.2%)	0.098^7^
No with peak ALT ≥ 5 × ULN and < 10 × ULN (%*n*^3^)	13 (2.0%)	16 (2.4%)	11 (1.7%)	0.656^7^
Bilirubin > 2 × ULN	1 (0.2%)	2 (0.3%)	1 (0.2%)	0.792^7^
INR^4^ >1.5	0 (0.0%)	0 (0.0%)	0 (0.0%)	
No with peak ALT ≥10 × ULN (%*n*^3^)	7 (1.1%)	2 (0.3%)	3 (0.5%)	0.164^7^
Bilirubin > 2 × ULN	2 (0.3%)	0 (0.0%)	1 (0.2%)	0.360^7^
INR^4^ >1.5	0 (0.0%)	0 (0.0%)	0 (0.0%)	
No of liver-related withdrawals	11 (1.7%)	7 (1.1%)	4 (0.6%)	0.178

Median days from the start of treatment to reach individual patient peak concentrations and the number of patients withdrawing from treatment for liver-related reasons are reported

*ALT* alanine aminotransferase, *INR* international normalised ratio, *IQR* interquartile range, *ULN* upper limit of normal

^1^ Some patients not included due to missing ALT results

^2^ If peak value ≥3 × ULN

^3^%*n* refers to percentage of total patients in the treatment arm

^4^ Patients with known anti-coagulant use were excluded from INR numbers

^5^ Isoniazid arm against standard therapy

^6^ Ethambutol arm against standard therapy

^7^ Chi-squared test (Fisher’s exact if any *n* ≤ 5)

### Cases of DILI and clinically significant elevations

A total of 58 of 1928 (3.0%) patients met the criteria for DILI defined in this analysis. This was made up of 22 of 639 (3.4%) patients taking standard therapy, 22 of 654 (3.4%) patients from the isoniazid arm and 14 of 634 (2.2%) patients on the ethambutol arm (chi-squared *p* = 0.34). A total of 101 of 1928 (5.2%) patients were considered to have clinically significant ALT elevations of ≥3 × ULN. Across the treatment arms, this was accounted for by 41 of 639 (6.4%) patients taking standard TB therapy, 35 of 654 (5.4%) patients on the isoniazid arm and 25 of 634 (3.9%) patients on the ethambutol arm (chi-squared *p* = 0.13).

Among the 22 patients who met the criteria for DILI on the standard therapy arm, eight patients reported diarrhoea and/or vomiting, ten reported nausea and three reported abdominal pain within 2 weeks of their peak ALT value. Five patients reported diarrhoea and/or vomiting and five patients had nausea among the DILI patients on the isoniazid arm. Among DILI cases on the ethambutol arm, two patients reported diarrhoea and/or vomiting, one patient experienced nausea and two patients complained of abdominal pain. Out of the 101 patients with clinically significant ALT elevations, 11 patients complained of diarrhoea and/or vomiting, ten reported nausea and one reported abdominal pain.

### Additional hepatotoxic factors and causality assessment

The trial protocol did not mandate that viral hepatitis serology be checked in every instance of clinically significant liver enzyme elevations and a majority of the patients were not tested for hepatitis E serology. However, the trial medical monitors strongly encouraged that this was performed in all these cases. Serology results indicating acute hepatitis B infection were found in two DILI cases (one on standard therapy and one on the ethambutol arm). Excess alcohol use was reported in eight cases meeting the criteria for clinically significant ALT elevations and DILI: three patients from the standard arm, one patient from the isoniazid arm and four patients from the ethambutol arm. There was one confirmed instance of herbal medication use in a DILI patient on the ethambutol arm.

The RUCAM scoring system was applied to 18 patients with DILI. The overall median value for the score was 8 out of 15 (interquartile range, IQR 4–9). The median values were 8 (IQR 3–8), 10.5 (IQR 3.5–13) and 7 (IQR 4.5–8.5) for DILI cases on the standard, isoniazid and ethambutol arms, respectively.

We identified one patient with Child–Pugh stage B liver disease at the time of their randomisation onto the isoniazid arm. This 24-year-old female patient was also HIV positive and not receiving anti-retroviral therapy. According to the database, the patient did not experience a recorded ALT or AST elevation >1 × ULN; however, she died after 45 days of treatment from ‘unclear abdominal symptoms’ with no death certificate issued.

### Timing of liver enzyme elevations

**Fig. 1:**
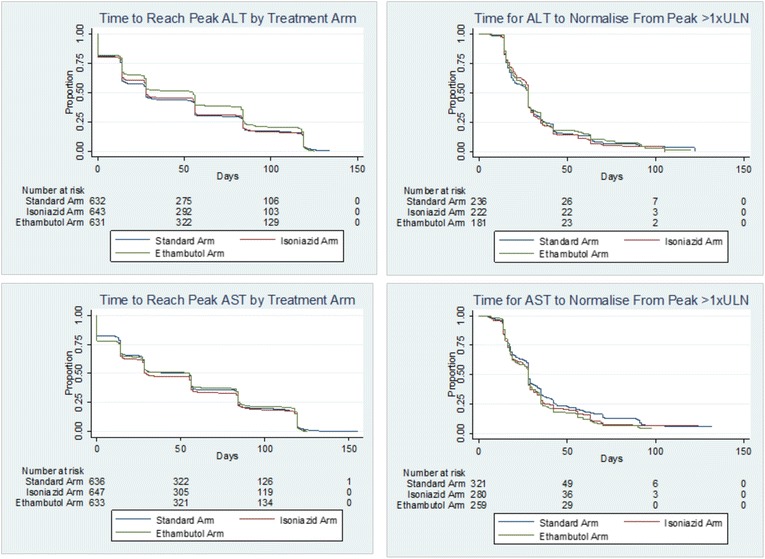
Kaplan–Meier curves for time to reach peak in all patients and normalisation of elevated ALT and AST values for patients with peak ≥1 × ULN. The curves for the time to reach peak ALT and AST show for all patients the time to reach the peak enzyme level by treatment arm. The log-rank test detected a significant difference for the time to reach peak ALT between the standard arm (median time 28 days) and ethambutol arm (median time 55 days). The time for patients with peak ALT and AST > 1 × ULN to return to within the normal range from this peak is also illustrated, with no significant difference detected between the treatment arms (*p* > 0.10). Eleven patients were not included in the time to peak graphs, as the peak value was measured at the screening visit (visit 0). ALT alanine aminotransferase, AST aspartate aminotransferase, ULN upper limit of normal

The median timing for ALT elevations can be seen in Table 1 and the Kaplan–Meier curves in Fig. 1 demonstrate the time to peak ALT and AST levels for all patients in the trial by treatment arm. There was no statistically significant difference on comparing the experimental and standard arms for time to peak and for time to normalise from a peak >1 × ULN (log-rank test, all *p* values > 0.100).

At the time of randomisation, 183 patients had a measured ALT > 1 × ULN and < 3 × ULN (65 patients from the standard arm, and 63 and 55 in the isoniazid and ethambutol arms). Of these 183 patients, 11 (6%) went on to meet the criteria for DILI on treatment (four patients taking standard therapy, and four and three patients allocated to the isoniazid and ethambutol arms).

Of the 1917 peak ALT measurements that were included in our analysis, a total of 38 were performed at unscheduled visits carried out based on the clinical judgement of a local physician. The median time for DILI was 28 days (IQR 14–56), and 21 of the 58 (36.2%) cases were measured at unscheduled visits. Furthermore, 25 of the 101 (24.8%) clinically significant elevations were identified at unscheduled visits at a median time of 25 days (IQR 14–56).

### Enzyme elevations in patients receiving isoniazid

The proportion of patients with ALT and/or AST ≥ 3 × ULN was significantly higher in isoniazid-containing arms (112 of 1293, 8.7%) than the ethambutol arm (38 of 635, 6.0%; *p* = 0.039). Similar percentages of patients had peak ALT ≥ 5 × ULN: 3% in both the standard and isoniazid arms and 2% in the ethambutol arm (see Table 1).

**Fig. 2:**
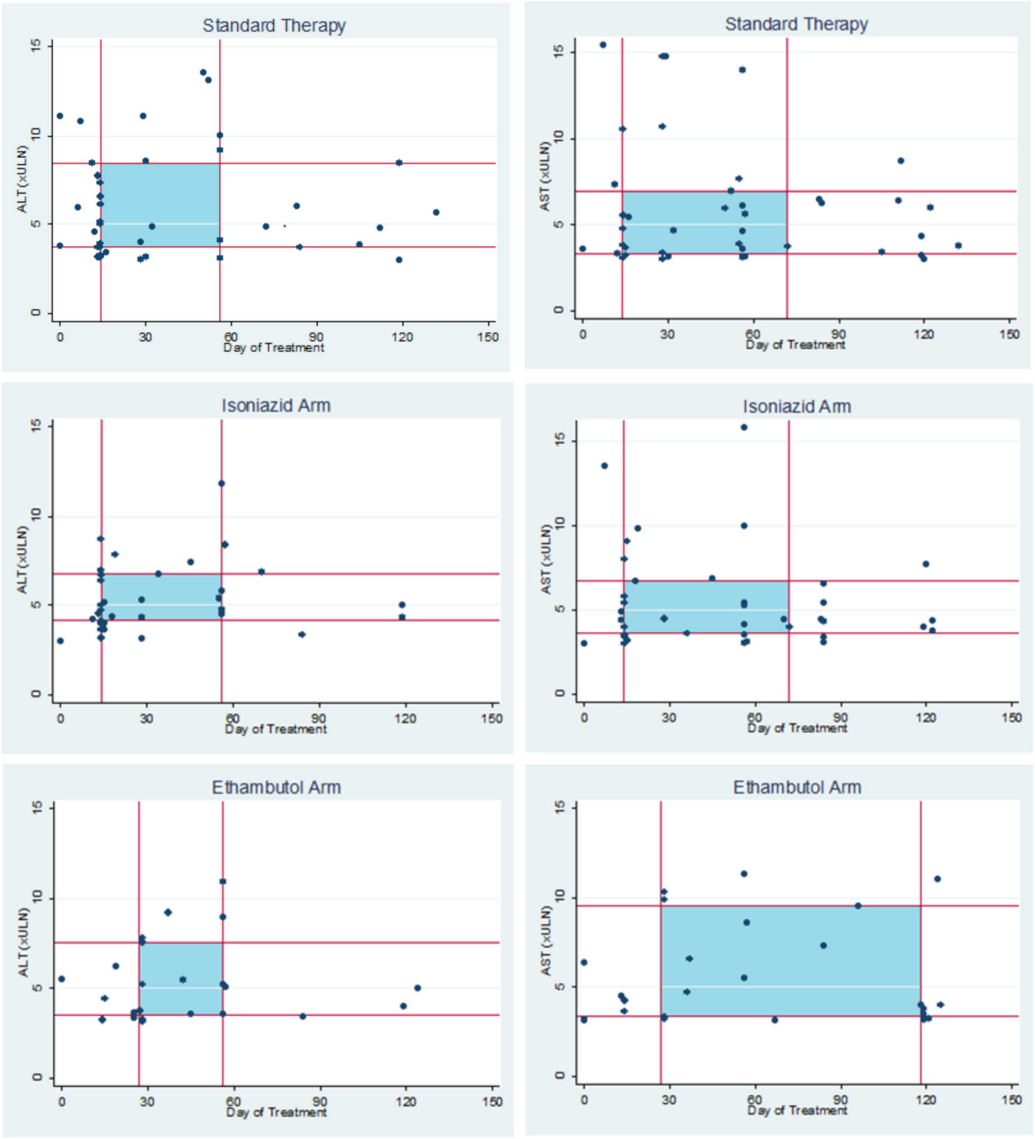
Scatter plots illustrating peak values for ALT and AST in patients when peak value ≥3 × ULN. The timing in days since the first treatment dose (*x*-axis) and peak ALT and AST (*y*-axis) is illustrated for each treatment arm for those patients with a peak ≥3 × ULN. The lines on the graphs indicate the interquartile ranges for the peak ALT and AST and the timing of the peak in this subgroup, with shaded areas corresponding to the interquartile range for both time and elevation result. Four patients excluded with ALT > 19 × ULN and six patients were excluded with AST >21 × ULN. ALT alanine aminotransferase, AST aspartate aminotransferase, ULN upper limit of normal

Patients in the ethambutol arm reached a peak ALT ≥ 3 × ULN 9.5 days later than patients receiving the isoniazid-containing regimens combined (median timing 28 days vs 18.5 days, *p* = 0.07). The median time to peak ALT concentration in all patients was significantly shorter in the two isoniazid-containing arms combined (28 days vs 55 days, *p* = 0.004). The relationship between peak value and the time that the peak occurred is displayed in Fig. 2 for patients with a peak value ≥3 × ULN in all treatment arms.

### Association of liver enzyme results with patient characteristics

Table 2 shows the prevalence of patients in each treatment arm who experienced a peak ALT or AST (hereafter ALT/AST) ≥3 × ULN while taking their allocated treatment, by subgroups defined by baseline characteristics. In the standard arm, the highest proportion of patients with a peak ALT/AST ≥ 3 × ULN was found in those aged >55 years with 8 of 38 patients (21.1%, *p* = 0.01).

**Table 2:** Relationship between elevation in liver enzyme concentration and patient characteristics at baseline by treatment regimen

	Peak ALT or AST ≥3 × ULN^1^		
	Standard arm (2EHRZ/4HR)	Isoniazid arm (2MHRZ/2MHR)	Ethambutol arm (2EMRZ/2MR)
*n* (%)	60 / 639 (9.4%)	52 / 654 (8.0%)	38 / 634 (6.0%)
Male (%)	40 / 447 (9.0%)	38 / 449 (8.5%)	24 / 447 (5.4%)
Female (%)	20 / 192 (10.4%)	14 / 205 (6.8%)	14 / 188 (7.5%)
Age in years^2^ (%)			
18–24	13 / 186 (7.0%)	17 / 203 (8.4%)	11 / 172 (6.4%)
25–34	21 / 184 (11.4%)	11 / 213 (5.2%)	9 / 210 (4.3%)
35–44	16 / 142 (11.3%)	11 / 115 (9.6%)	8 / 122 (6.6%)
45–54	2 / 88 (2.3%)	6 / 81 (7.4%)	8 / 86 (9.3%)
≥55	8 / 38 (21.1%)	7 / 41 (17.1%)	2 / 44 (4.6%)
Baseline weight in kg (%)			
<40	11 /63 (17.5%)	6 / 56 (10.7%)	5 / 56 (8.9%)
40–49	22 / 218 (10.1%)	20 / 243 (8.2%)	11 / 224 (4.9%)
50–59	16 / 242 (6.6%)	19 / 231 (8.2%)	12 / 236 (5.1%)
60–69	8 / 88 (9.1%)	5 / 88 (5.7%)	8 / 86 (9.3%)
≥70	3 / 28 (10.7%)	2 / 36 (5.6%)	2 / 33 (6.1%)
Ethnicity (%)			
Black	20 / 295 (6.8%)	21 / 277 (7.6%)	17 / 290 (5.9%)
Asian	34 / 194 (17.5%)	23 / 201 (11.4%)	10 / 193 (5.18%)
Mixed race	6 /149 (4.0%)	8 / 174 (4.6%)	11 / 151 (7.3%)
Other	0 / 1 (0.0%)	0 / 2 (0.0%)	0 / 0 (0.0%)
HIV positive (%)	7 / 46 (15.2%)	3 / 46 (6.5%)	4 / 48 (8.3%)
HIV negative (%)	53 / 593 (8.9%)	49 / 608 (8.1%)	34 / 586 (5.8%)
Smoking history (%)			
Never	31 / 298 (10.4%)	23 / 291 (7.9%)	16 / 279 (5.7%)
Previous	11 / 155 (7.1%)	17 / 155 (11.0%)	9 / 166 (5.4%)
Current	18 / 186 (9.7%)	12 / 208 (5.8%)	13 / 190 (6.8%)

Characteristics for patients across all treatment arms are shown according to peak ALT and/or AST while taking treatment

*ALT* alanine aminotransferase, *AST* aspartate aminotransferase, *ULN* upper limit of normal

^1^ Cell percentages shown

^2^ Data for date of birth was not available for some patients in all three treatment arms

Seven of 46 (15.2%) HIV-positive patients taking standard therapy had elevated ALT/AST ≥ 3 × ULN compared to 53 of 593 HIV-negative patients (8.9%) but this difference was not significant (*p* = 0.160).

Significantly more Asian patients experienced ALT/AST ≥ 3 × ULN compared to other ethnic groups [11.4% (Asian) vs 6.7% (black) vs 5.5% (mixed race), *p* < 0.001]. Also, a significantly higher proportion of Asian patients experienced DILI (28 of 560, 4.8%) compared to the other ethnic groups (2.0% black, 0% Caucasian, and 2.7% mixed race and other; *p* = 0.02). Of the 67 Asian patients with a peak ALT/AST ≥ 3 × ULN, 57 (85.1%) were taking isoniazid-containing regimens and 10 (14.9%) were allocated to the ethambutol arm (*p* = 0.008).

### Patients with extreme peak enzyme results

Seven of 639 (1.1%) on standard therapy exhibited a peak ALT ≥ 10 × ULN (range 10.1–19.1 × ULN), and among these patients, the median time to peak ALT was 29 days of therapy (IQR 7–52). Six of these patients were male, the median age was 33.3 years (IQR 28.5–40.9 years) and two patients were HIV positive. Alcohol use was confirmed as an underlying feature in two cases, and one of these patients had positive serology for acute hepatitis B. Most patients (5/7) reported nausea or vomiting, and one reported abdominal pain within 2 weeks of the peak elevation.

Two patients in the isoniazid arm had peak ALT ≥ 10 × ULN (39.6 × ULN with bilirubin 1.7 × ULN and 11.9 × ULN at 17 and 56 days, respectively). Both were HIV negative and less than 30 years old. No symptoms were recorded for either of these patients.

In the ethambutol arm, three patients developed peak ALT elevations of ≥10 × ULN (range 11.0–38.8 × ULN) with a range of 27–56 days. The median age of the three patients was 42.5 years (range 39.5–49.5 years). There was one HIV-positive patient assigned to the ethambutol arm, and the patient was not receiving anti-retroviral therapy. This patient, for whom no predisposing conditions were identified, suffered a severe and fatal course. Evidence of DILI arose after 2 weeks of treatment and they exhibited haemodynamic compromise, a maculopapular rash and symptoms of gastroenteritis. ALT peaked at 38.8 × ULN and total bilirubin at 6.2 × ULN. No post-mortem was performed, with cause of death pronounced as ‘hepatitis of unknown cause’.

### Withdrawals due to liver toxicity

Out of 43 total deaths in the REMoxTB trial, only two were linked to hepatotoxicity, as reported above. Only 22 patients were withdrawn from the trial due to liver-related events (see Table 1), and none of these patients died. Their mean age was 37 years (±13.96 years) and 73% were male. The median ALT peak in this group was 7.09 × ULN (IQR 2.83–10.05) occurring at 34.5 days, and the median peak AST was 5.99 × ULN (IQR 3.03–11.03) at 28.5 days. Of 22 patients, 10 (45.5%) had symptoms recorded; nine with diarrhoea and/or vomiting, six reported nausea and one patient developed mild jaundice. Seventeen of the 22 (77.3%) patients had achieved a microbiological cure at 18 months, after onward referral to the National Treatment Programme.

## Discussion

In this paper, we have shown that 94% of patients completed their course of standard TB therapy without clinically significant ALT elevations detected. There were similar proportions of 95% in the isoniazid arm and 96% in the ethambutol arm.

There has been an assumption that the majority of elevations during TB treatment will occur within the first 2 to 4 weeks of therapy. One recent paper reported that over half of the patients who developed elevated liver enzymes within 2 weeks of starting treatment had good specificity for predicting hepatotoxicity later during treatment using a monitoring schedule of regular testing for the first 2 weeks of treatment [16]. The BTS and ATS [6, 13] both recommend pre-treatment baseline blood tests followed by clinical assessment in all patients. In patients deemed by the physician to be at higher risk of DILI or with existing liver disease or age > 35 years old, the ATS recommends a further blood test between 2 and 4 weeks after initiating therapy [6]. The BTS advises a policy of further weekly blood tests for 2 weeks and 2-weekly blood tests for the first 2 months for patients with known liver dysfunction. Current guidance from the World Health Organization does not include regular monitoring for hepatotoxicity, and instead symptoms are used to detect liver dysfunction [21].

In REMoxTB, the IQR for the time to reach peak ALT ≥ 3 × ULN was 14–56 days for patients taking standard TB therapy, suggesting that 75% of these patients would have been detected by a regular LBT during the first 60 days of treatment. Based on the pattern of elevations observed, we would recommend a monitoring schedule consisting of LBTs at baseline, 1 week (to identify early elevators), 2 weeks (lower end of the IQR), 4 weeks (median timing for elevations) and 8 weeks (upper end of the IQR). We suggest that this schedule is achievable even in resource-poor settings and that our findings suggest diminishing return for LBTs after 8 weeks of therapy in the absence of symptoms.

There are unanswered questions surrounding the significance of isolated ALT elevations regarding how best to evaluate these patients, and which are the most appropriate biochemical markers to be included in the evaluation of ALT elevations [22]. Isolated liver enzyme levels on treatment could reflect hepatic adaptation to the medication, wherein transient LBT abnormalities resolve even with continued exposure to the drug and the patient remains clinically well throughout [23]. One large cohort study of over 11,000 patients receiving isoniazid preventive therapy observed symptoms and signs of hepatotoxicity in only 0.15% of patients completing therapy [24], suggesting that the rates of DILI/enzyme elevations requiring intervention are actually very low with monotherapy and, by extension, with standard TB therapy as well. Certainly, in our study a minority of patients with DILI or clinically significant elevations had recorded symptoms or bilirubin elevations and in the trial, the majority of patients who experienced liver enzyme elevations went on to be cured of their disease.

A prospective series of 1223 patients with drug-induced liver failure demonstrated that TB therapy-induced liver failure can often have a subacute presentation and was associated with 67% mortality [25]. The fatality seen on the ethambutol arm involving an ALT rise of 38 × ULN highlights the risk of mortality for patients presenting with hyperbilirubinaemia and elevated levels of liver enzymes. While the local physician evaluated this as ‘hepatitis of unknown cause’, it would seem reasonable to consider this as DILI. By comparison, the mortality associated with acute viral hepatitis is low and the majority of deaths are accounted for by the chronic sequelae of the disease [26].

Despite most patients being clinically unaffected by ALT elevations on treatment, we still demonstrated an overall risk of 3.4% for DILI on standard TB therapy and higher rates of DILI among those patients with enzyme elevations at randomisation compared with the overall trial population. Given the challenging circumstances surrounding TB treatment (with much higher levels of HIV co-infection than seen in REMoxTB), we would still recommend the conservative monitoring schedule laid out above to allow for the early detection of at-risk patients. Given that the majority of patients with liver enzyme elevations were not withdrawn, there is some evidence in favour of attempting to re-introduce the same regimen following a treatment interruption, as opposed to immediately changing to a more liver-sparing regimen.

An association between significant hepatic events secondary to TB treatment and HIV-positive status has been reported previously [6, 27–29]. However, the precise magnitude of this risk is not clear. The observed higher rate of ALT/AST elevations in HIV-positive individuals in this study is similar to that in other reports [27–29], although this did not achieve statistical significance in our study. Lower CD4+ counts have been significantly associated with risk for sub-clinical hepatotoxicity in HIV-positive individuals on TB therapy [30]. Exclusion from the trial of patients with CD4 < 250 cells/μL may have reduced the rates of hepatotoxicity.

Our data support the increased risk of hepatotoxicity in certain ethnic groups [31, 32], and in our study, we found evidence to support a higher risk of liver enzyme elevations in Asian patients. Polymorphisms affecting the cytochrome P450 and NAT2 liver enzymes are associated with an increased risk of hepatotoxicity, and NAT2 is especially related to the metabolism of isoniazid [33–35]. However, it needs to be remembered that there may be factors unique to individual sites that confound the relationship between ethnicity and liver function changes, such as alcohol use and viral hepatitis. Additionally, although based on small numbers, we have seen a higher proportion of older patients experiencing enzyme elevations when taking standard TB therapy.

After observing unacceptably high rates of significant hepatotoxicity associated with the use of rifampicin and pyrazinamide to treat latent TB infection [36, 37], a protective effect of isoniazid on the liver was hypothesised by some as a possible explanation [38, 39]. In REMoxTB, those patients taking isoniazid-containing regimens exhibit significantly higher and earlier peak ALT and AST values on treatment. Combined with the significantly lower peak values seen in the moxifloxacin-containing arms (lowest in the ethambutol arm), this supports the use of moxifloxacin in liver-sparing regimens. Specifically, the low rate of enzyme elevations in the ethambutol arm could suggest a role for this as a liver-sparing regimen, but more work is needed to investigate the ideal duration [17].

Clinical trials are not fully representative of the diversity of TB patients as CD4 and liver enzyme criteria were set for admission. Early in the REMoxTB study, only AST was measured, and not ALT, which led to absent ALT results for some patients. The study protocol did not call for alkaline phosphate measurements and we were, therefore, unable to apply fully the criteria for Hy’s law to clinically significant enzyme elevations. The timings of temporary medication pauses were also not recorded with sufficient accuracy in the database.

## Conclusions

In conclusion, we show that older age (on standard therapy), Asian ethnicity and the presence of isoniazid in a treatment regimen are significantly associated with liver enzyme elevations during treatment. Closer monitoring of these patients during treatment may be beneficial, and there is justification for the exclusion of isoniazid from liver-sparing regimens. Despite relatively high rates of liver enzyme elevations, the majority of patients completed the course of their treatment with a high cure rate in the standard therapy. Standard TB therapy is lengthy and toxic, and this work further emphasises the need to develop shorter, effective and better-tolerated treatment to combat the ongoing global epidemic of TB.

## Additional file

Additional file 1: **Table S1.** Daily dosing of TB medications for patients randomised into REMoxTB based on weight at screening. **Table S2.** The Roussel–Uclaf causality assessment method (RUCAM) for causality assessment of adverse drug reactions. **Table S3.** Child–Pugh scoring system for grading the prognosis of chronic liver disease. **Figure S1.** Graphs showing the median ALT and AST values for all patients at scheduled blood draws in all three treatment arms. (DOCX 318 kb)
