# Supplementary material for: Liver toxicity associated with tuberculosis chemotherapy in the REMoxTB study
Source: BMC Med. 2018 Mar 28;16:46. doi: 10.1186/s12916-018-1033-7 (PMC5875008; doi:10.1186/s12916-018-1033-7)
Supplement: Supplementary file 1 — Table S1. Daily dosing of TB medications for patients randomised into REMoxTB based on weight at screening. Table S2. The Roussel–Uclaf causality assessment method (RUCAM) for causality assessment of adverse drug reactions. Table S3. Child–Pugh scoring system for grading the prognosis of chronic liver disease. Figure S1. Graphs showing the median ALT and AST values for all patients at scheduled blood draws in all three treatment arms. (DOCX 318 kb) [file 12916_2018_1033_MOESM1_ESM.docx]

| **Drug Name** | | **Daily Dosing** |
| --- | --- | --- |
| Moxifloxacin | | 400mg |
| Rifampicin | |  |
|  | <45 kg | 450mg |
|  | >45 kg | 600mg |
| Isoniazid | | 300mg |
| Pyrazinamide | |  |
|  | <40 kg | 25mg/kg (rounded to nearest 500mg) |
|  | 40 - 55 kg | 1000mg |
|  | >55 – 75 kg | 1500mg |
|  | >75 kg | 2000mg |
| Ethambutol | |  |
|  | <40 kg | 15mg/kg (rounded to nearest 100mg) |
|  | 40 - 55 kg | 800mg |
|  | >55 – 75 kg | 1200mg |
|  | >75 kg | 1600mg |

**Table S1: Daily dosing of TB medications for patients randomised into REMoxTB based on weight at screening.** Isoniazid and moxifloxacin were both given at fixed doses irrespective of weight

| **Criteria** | | **Score** |
| --- | --- | --- |
| Time to onset of the reaction | |  |
|  | Highly suggestive | +3 |
|  | Suggestive | +2 |
|  | Compatible | +1 |
|  | Inconclusive | 0 |
| Course of the reaction | |  |
|  | Highly suggestive | +3 |
|  | Suggestive | +2 |
|  | Compatible | +1 |
|  | Against the role of the drug | -2 |
|  | Inconclusive or unavailable | 0 |
| Risk factor(s) for drug reaction | |  |
|  | Presence | +1 to +2 |
|  | Absence | 0 |
| Concomitant drugs | |  |
|  | Time to onset incompatible | 0 |
|  | Time to onset compatible, but unknown reaction | -1 |
|  | Time to onset compatible and known reaction | -2 |
|  | Role proved in this case | -3 |
|  | None or no information available | 0 |
| Non-drug related causes | |  |
|  | Ruled out | +2 |
|  | Possible or not investigated | +1 to -2 |
|  | Probable | -3 |
| Previous information on the drug | |  |
|  | Reaction unknown | 0 |
|  | Reaction published but unlabelled | +1 |
|  | Reaction labelled in product characteristics | +2 |
| Response to readministration | |  |
|  | Positive | +3 |
|  | Compatible | +1 |
|  | Negative | -2 |
|  | Not available or uninterpretable | 0 |

**Table S2: The Roussel-Uclaf Causality Assessment Method (RUCAM) for causality assessment of adverse drug reactions**

| **Measure** | **1 point** | **2 points** | **3 points** |
| --- | --- | --- | --- |
| [Total bilirubin](https://en.wikipedia.org/wiki/Bilirubin), μmol/L (mg/dL) | <34 (<2) | 34–50 (2–3) | >50 (>3) |
| [Serum albumin](https://en.wikipedia.org/wiki/Serum_albumin), g/dL | >3.5 | 2.8–3.5 | <2.8 |
| [Prothrombin time](https://en.wikipedia.org/wiki/Prothrombin_time), prolongation (s) | <4.0 | 4.0–6.0 | > 6.0 |
| [Ascites](https://en.wikipedia.org/wiki/Ascites) | None | Mild (or suppressed with medication) | Moderate to severe (or refractory) |
| [Hepatic encephalopathy](https://en.wikipedia.org/wiki/Hepatic_encephalopathy) | None | Grade I–II | Grade III–IV |

**Table S3: Child-Pugh scoring system for grading the prognosis of chronic liver disease.** Class A: 5-6 points (85% 2 year survival); Class B: 7-9 points (57% 2 year survival); Class C: 10-15 points (35% 2 year survival)

**Figure S1: Graphs showing the median ALT & AST values for all patients at scheduled blood draws in all three treatment arms**
